# Prevalence and predictors of no-shows to physical therapy for musculoskeletal conditions

**DOI:** 10.1371/journal.pone.0251336

**Published:** 2021-05-28

**Authors:** Nrupen A. Bhavsar, Shannon M. Doerfler, Anna Giczewska, Brooke Alhanti, Adam Lutz, Charles A. Thigpen, Steven Z. George

**Affiliations:** 1 Division of General Internal Medicine, Department of Medicine, Duke University School of Medicine, Durham, NC, United Stated of America; 2 Duke Clinical Research Institute, Duke University, Durham, NC, United Stated of America; 3 ATI Physical Therapy, Greenville, SC, United Stated of America; 4 Exercise Science, Arnold School of Public Health, University of South Carolina, Columbia, SC, United Stated of America; 5 Center for Effectiveness Research in Orthopaedics, Arnold School of Public Health, University of South Carolina, Columbia, SC, United Stated of America; 6 Department of Orthopaedic Surgery, Duke University School of Medicine, Durham, NC, United Stated of America; Western University, CANADA

## Abstract

**Objectives:**

Chronic pain affects 50 million Americans and is often treated with non-pharmacologic approaches like physical therapy. Developing a no-show prediction model for individuals seeking physical therapy care for musculoskeletal conditions has several benefits including enhancement of workforce efficiency without growing the existing provider pool, delivering guideline adherent care, and identifying those that may benefit from telehealth. The objective of this paper was to quantify the national prevalence of no-shows for patients seeking physical therapy care and to identify individual and organizational factors predicting whether a patient will be a no-show when seeking physical therapy care.

**Design:**

Retrospective cohort study.

**Setting:**

Commercial provider of physical therapy within the United States with 828 clinics across 26 states.

**Participants:**

Adolescent and adult patients (age cutoffs: 14–117 years) seeking non-pharmacological treatment for musculoskeletal conditions from January 1, 2016, to December 31, 2017 (n = 542,685). Exclusion criteria were a primary complaint not considered an MSK condition or improbable values for height, weight, or body mass index values. The study included 444,995 individuals.

**Primary and secondary outcome measures:**

Prevalence of no-shows for musculoskeletal conditions and predictors of patient no-show.

**Results:**

In our population, 73% missed at least 1 appointment for a given physical therapy care episode. Our model had moderate discrimination for no-shows (c-statistic:0.72, all appointments; 0.73, first 7 appointments) and was well calibrated, with predicted and observed no-shows in good agreement. Variables predicting higher no-show rates included insurance type; smoking-status; higher BMI; and more prior cancellations, time between visit and scheduling date, and between current and previous visit.

**Conclusions:**

The high prevalence of no-shows when seeking care for musculoskeletal conditions from physical therapists highlights an inefficiency that, unaddressed, could limit delivery of guideline-adherent care that advocates for earlier use of non-pharmacological treatments for musculoskeletal conditions and result in missed opportunities for using telehealth to deliver physical therapy.

## Introduction

Musculoskeletal (MSK) pain (e.g., of the back, neck, or extremity) is one of the, if not the, largest subgroups of chronic pain conditions [1, 2] and frequently results in opioid prescriptions [3]. Physical therapy is a commonly used non-pharmacological approach for MSK conditions and includes a variety of front-line treatments for chronic pain recommended by clinical practice guidelines [1, 4, 5]. Observational studies indicate that receiving physical therapy early in a treatment episode limits chronic opioid use for many MSK conditions [6–10]. Accordingly, early delivery of physical therapy services for MSK conditions may be a key component of providing guideline-adherent care and reducing excessive health care utilization for chronic MSK conditions [11, 12].

Out of the 50 million people impacted by chronic pain, only 12 million people in the United States accessed outpatient physical or occupational therapy [13]. Across common MSK conditions, like low back pain and rotator cuff tear, physical therapy utilization ranges from 3–10% with significant variation across health referral regions [14–16]. Utilization is limited for physical therapy services in part by the available workforce. It is estimated that the number of therapists is 10–20% less than the number of patients in need [17]. This limitation in the available physical therapy workforce is a significant long-term barrier to health care systems increasing rates of non-pharmacological care provided by physical therapists. In the short-term, novel approaches that utilize the current workforce are needed to improve the scale of physical therapy care for MSK conditions.

One such opportunity is through better use of already scheduled physical therapy appointments. Efficiency in care delivery decreases as patients do not appear for scheduled appointments–termed “no-shows.” While these “no-shows” (termed “did not attend” or “unable to attend” in other settings) are often attempted to be rescheduled, the available clinician time is lost with no way to recapture that care opportunity. No-shows are associated with decreased efficiencies including wasted resources (e.g., clinician time and decreased revenue), inadequate dosing of therapy for current patients, and delay in care initiation for other patients [18]. These inefficiencies result in large monetary and non-monetary costs associated with patient no-shows. One large family practice center estimated a no-show rate of 31% among 45,000 patients per year; this translated into a total annual revenue shortfall of 3–14%. Only two small (<800 patients total) studies have reported no-show rates for physical and occupational therapy to range between 11–31% in Australia and United Kingdom [19, 20]. However, we are not aware of any published data describing the degree or impact of no-shows on physical therapy delivery in the United States. Limiting no-shows for non-pharmacological providers is an important topic to consider for health care systems because it provides potential benefits of enhancement of workforce efficiency without growing the existing provider pool, delivering guideline adherent care, and identifying those that may benefit from telehealth.

In this study, we used a database of patients receiving physical care for MSK conditions from a large United States commercial provider. Our goals were to 1) describe the national prevalence of no-shows and 2) develop and validate predictive algorithms for patient no-shows to physical therapy care. In the analyses, we distinguished between operative and non-operative care episodes due to inherent differences in the way their care is structured.

## Materials and methods

### Design and data

The study data are from patients seeking care for chronic pain at the largest, national provider of outpatient physical therapy with 828 clinics across 26 states in the United States. The company employs over 2500 physical therapists who provide care to over 400,000 unique patients every year representing approximately 4% of the outpatient physical therapy market. In 20 of 26 states where clinics were located, patients have direct access (i.e., no referral necessary). However, most insurance companies do require a referral. Once a referral has been submitted, patients schedule their preferred day and time for their appointment. After patients attend their first visit and initiate care, information from the encounter is documented within the electronic health record (EHR) their data and is then available from a Patient Outcomes Registry.

Patients were eligible for inclusion in this study if greater than or equal to 14 years and less than or equal to 116 years of age with at least one scheduled visit for an episode of care from January 1, 2016, to December 31, 2017 (n = 542,685). The upper and lower ages were selected based on the expected minimum of those routinely seeking care in those clinics (i.e., atypical for those younger than 14 to be seen in that setting) and the maximum age of what is clinically feasible (i.e., oldest age expected). Patients were excluded if they had missing or outlying values for age, height, weight, or BMI (height less than 24 inches or greater than 84 inches, weight less than 50 pounds or greater than 400 pounds, or body mass index (BMI) less than 10 or greater than 75). This resulted in 112346 exclusions. After exclusions, 444,995 patients across 697 clinics in 26 states were included in the analyses (S1 Fig).

### Outcome assessment

The primary outcome was patient no-show defined by a cancellation or no appearance without previously cancelling a scheduled appointment across all visits. A no-show in these data was defined as a visit that was scheduled but had a final status of "canceled" or "pending" instead of "completed." A “pending” status indicated the patient had a scheduled appointment but did not cancel or complete that appointment with a visit.

### Predictor variables

**Fig 1:**
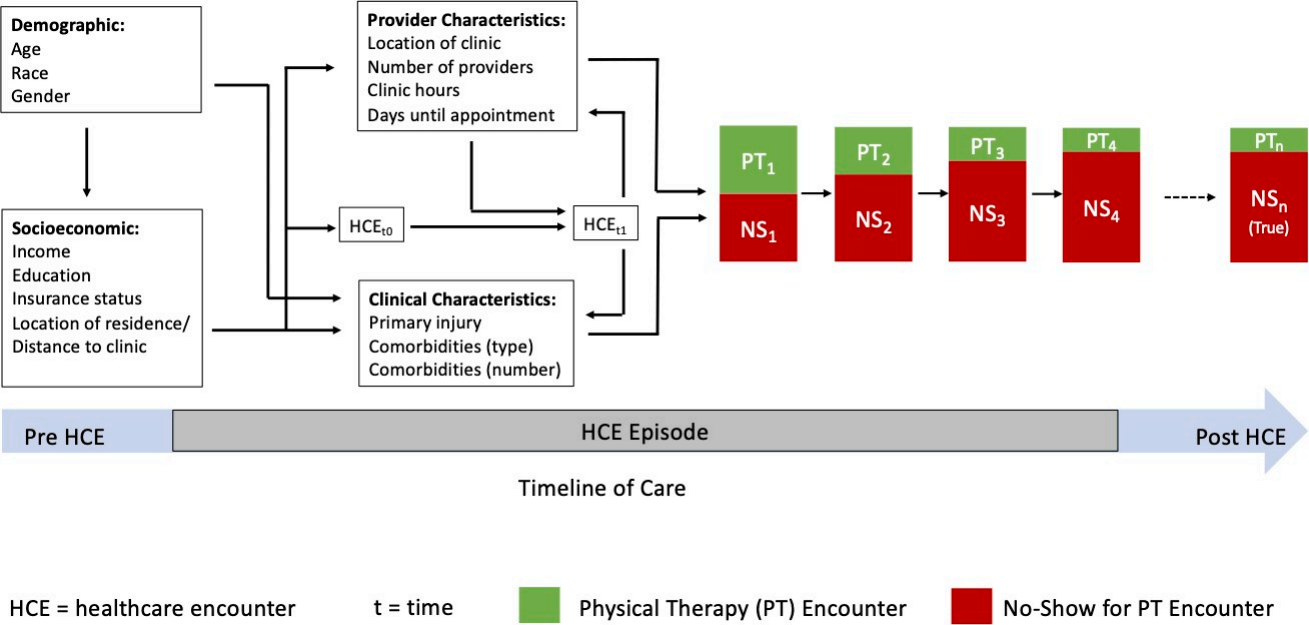
Multi-level factors associated with no-shows in physical therapy clinics.

The goal of this project was to develop a risk score that can be implemented to decrease “no-show” rates. When designing the study, we consulted with clinical providers, administrators, and the literature [21–24] to extract variables that may predict no-shows. We summarized these results into a conceptual model (Fig 1) of the relationship between these variables and the probability of a patient not showing for an appointment. The figure shows that demographic characteristics can impact socioeconomic factors such as location of residence, and clinical characteristics such as the type and number of comorbidities. Independently, socioeconomic factors can impact provider characteristics (e.g., provider seen), clinical characteristics, and the probability of a healthcare encounter. Both, the initial healthcare encounter and provider characteristics impact the probability of a future healthcare encounter. Provider characteristics, a healthcare encounter, and clinical characteristics impact the probability that a patient attends an appointment or is a no-show. Results from the current study show that prior no-shows increase the probability of subsequent no-show. After four days, the probability of a no-show stabilized, resulting in the true overall no-show probability for a patient. We added this information to our conceptual model a posteriori.

Based on this, we chose demographic, clinical, encounter-related, and organizational variables that predicted no-shows. Demographic variables included age, sex, and insurance status. Patient race/ethnicity was not uniformly collected during encounters and was therefore not included as an individual-level predictor. Clinical characteristics captured at the initial visit included primary body region for seeking care, report of pain or symptoms in a secondary body region, medical comorbidities, and symptom reports. Encounter characteristics included number of previously completed visits, number of cancellations, time from first to current visit, time from previous to current visit, time from when visit was scheduled to visit date, day of the week, cumulative number of visits, month of visit, and year of visit. Organizational predictors included state within which a clinic resides, full time equivalents (FTE, where 1 FTE = 40 hours patient care/week), and type of clinician (i.e., physical therapy or physical therapy assistant).

### Ethics statement

This study was determined to be exempt by the Institutional Review Board at Duke University (Pro00088901). Consent was not obtained because the data were retrospective administrative data and they were analyzed anonymously.

### Statistical analysis

#### Model development

The data were split into training and testing sets. The training data, used for model development, were obtained by randomly selecting 75% of the data. The remaining 25% were used as the independent test data to assess model performance. After considering different modeling approaches, we used least absolute shrinkage and selection operator (LASSO), a type of regularized logistic regression that performs feature selection and provides interpretable, parsimonious models. There are multiple advantages to using LASSO. It provides very good prediction accuracy by shrinking coefficients to reduce variance without a substantial impact on the bias. LASSO also helps to improve model interpretability by eliminating variables that are not associated with the outcome, reducing model overfit [25, 26]. We used ten-fold cross-validation to select the optimal LASSO model and further avoid overfitting.

The test dataset was used to evaluate the performance and validity of the model. Model calibration was assessed using the Brier score and calibration plots (i.e., logistic, nonparametric), which compared the predicted probability of a no-show with the observed probability. Model discrimination–defined as the ability of the model to discriminate between those who did and did not show for their appointment–was assessed using the concordance (*c*)statistic and Somers’s D statistic as well as plots of the area under the curve (AUC).

In addition to the overall models, we fit the models according to the patient’s operative status due to the large difference in physical therapy utilization for non-operative versus post-operative patients. We also considered different right truncation points based on the distribution of the number of visits. We fit models using the first visit, the first 7 cumulative visits, and the first 12 cumulative visits. The 7^th^ and 12^th^ visits were chosen as they corresponded to the 25^th^ and 50^th^ percentiles, respectively, of the number of visits. In sensitivity analyses, we examined the prediction characteristics of the model and factors predictive of a no-show stratified by age groups. We also examined the prediction characteristics of the model when insurance status was excluded from the set of potential predictors. Some insurance companies may have restrictions on the number of treatments allowed and it is possible that some payers may have only allowed for a single visit.

The study was conducted using the TRIPOD guidelines for model development and validation. All analyses were completed using R v3.4.2 (The R Foundation for Statistical Computing, Vienna, Austria) and SAS 9.4 (SAS Institute, Cary, NC). Packages in R used for this analysis include tidyverse, matrix, glmnet, rocr, hmisc, and lattice [27–34]. This study was determined to be exempt by the Institutional Review Board at Duke University.

#### Patient or public involvement

No patients involved.

## Results

### Study population

The study cohort for this analysis consisted of 444,995 patients and 6,023,363 encounters seen at one of the company’s physical therapy clinics in the United States. A majority of the population was female (59%) with a mean age of 53 years, mean BMI of 29 kg/m^2^, and used commercial insurance (58%) (Table 1). As expected, the body regions most commonly affecting patients were lumbar/SI (i.e., low back) (26%), knee (20%), and shoulder (16%). The most prevalent medical comorbidities reported at baseline were arthritis (37%), high blood pressure (33%), and breathing difficulties/asthma (15%). The most common symptoms reported at baseline were night sweats/night pain (16%) and ringing in ears (14%).

**Table 1:** Characteristics of the study population at initial visit.

	Overall N = 444,995	Non-operative N = 374,791	Post-operative N = 70,204
**Patient-level characteristics**			
**Age (years)**			
Mean (min, max)	53 (14, 117)	52 (14, 117)	55 (14, 99)
Median (Q1, Q3)	54 (39, 67)	54 (38, 66)	58 (45, 67)
**Female**	262906 (59.1%)	226794 (60.5%)	36112 (51.4%)
**Pregnant**	1749/262906 (0.7%)	1715/226794 (0.8%)	34/36112 (0.1%)
**Height (inches)**			
Mean (min, max)	67 (36, 84)	67 (36, 84)	67 (40, 84)
Median (Q1, Q3)	66 (64, 70)	66 (64, 70)	67 (64, 70)
**Weight (pounds)**			
Mean (min, max)	182 (54, 400)	180 (54, 400)	189 (60, 400)
Median (Q1, Q3)	175 (149, 208)	175 (146, 205)	184 (155, 215)
**BMI (kg/m**^**2**^**)**			
Mean (min, max)	29 (11, 75)	28 (11, 74)	29 (11, 75)
Median (Q1, Q3)	27 (24, 32)	27 (24, 32)	28 (25, 33)
**Ever smoker**	65275 (14.7%)	54795 (14.6%)	10480 (14.9%)
**Insurance provider**			
Commercial	257313 (57.8%)	214375 (57.2%)	42938 (61.2%)
Medicaid	44894 (10.1%)	40752 (10.9%)	4142 (5.9%)
Medicare	89760 (20.2%)	74087 (19.8%)	15673 (22.3%)
Other	8437 (1.9%)	7106 (1.9%)	1331 (1.9%)
Workers’ Comp	44591 (10.0%)	38471 (10.3%)	6120 (8.7%)
**Therapist type**			
PT	436007 (98.0%)	367442 (98.0%)	68565 (97.7%)
PTA	8988 (2.0%)	7349 (2.0%)	1639 (2.3%)
**Number of providers at the clinic**			
Mean (min, max)	2 (0, 15)	2 (0, 15)	2 (0, 15)
Median (Q1, Q3)	2 (1, 3)	2 (1, 3)	2 (1, 3)
**Clinical characteristics**			
**Primary body region**			
Elbow/Wrist/Hand	12900 (2.9%)	9859 (2.6%)	3041 (4.3%)
Foot/Ankle	42263 (9.5%)	33672 (9.0%)	8591 (12.2%)
General	765 (0.2%)	762 (0.2%)	3 (0.0%)
Hip	32275 (7.3%)	24246 (6.5%)	8029 (11.4%)
Knee	85874 (19.3%)	56034 (15.0%)	29840 (42.5%)
Lumbar/SI	116298 (26.1%)	110812 (29.6%)	5486 (7.8%)
Neck	55397 (12.4%)	53257 (14.2%)	2140 (3.0%)
Shoulder	70144 (15.8%)	57521 (15.3%)	12623 (18.0%)
Other	29079 (6.5%)	28628 (7.6%)	451 (0.6%)
Chronic injury	81354 (18.3%)	66717 (17.8%)	14637 (20.8%)
**Comorbidities**			
Arthritis	165354 (37.2%)	134490 (35.9%)	30864 (44.0%)
High blood pressure	145655 (32.7%)	119249 (31.8%)	26406 (37.6%)
Breathing difficulties/ Asthma	65205 (14.7%)	56692 (15.1%)	8513 (12.1%)
Diabetes	57127 (12.8%)	48147 (12.8%)	8980 (12.8%)
Heart condition	47506 (10.7%)	40231 (10.7%)	7275 (10.4%)
Osteoporosis	39395 (8.9%)	32574 (8.7%)	6821 (9.7%)
Cancer	36491 (8.2%)	30381 (8.1%)	6110 (8.7%)
Psychological condition	28134 (6.3%)	24805 (6.6%)	3329 (4.7%)
Chest painKidney condition	18458 (4.1%)	15999 (4.3%)	2459 (3.5%)
Stroke	14529 (3.3%)	12801 (3.4%)	1728 (2.5%)
**Symptom reports**			
Night sweats/ Night pain	71316 (16.0%)	62922 (16.8%)	8394 (12.0%)
Ringing in your ears	60499 (13.6%)	52574 (14.0%)	7925 (11.3%)
Fracture	43073 (9.7%)	33086 (8.8%)	9987 (14.2%)
Difficulty swallowing	14906 (3.3%)	12972 (3.5%)	1934 (2.8%)
**Number of comorbidities**			
Mean (min, max)	1 (0, 11)	1 (0, 11)	1 (0, 11)
Median (Q1, Q3)	1 (0, 2)	1 (0, 2)	1 (0, 2)
**Number of symptoms reported**			
Mean (min, max)	0 (0, 4)	0 (0, 4)	0 (0, 4)
Median (Q1, Q3)	0 (0, 1)	0 (0, 1)	0 (0, 1)
**Visits**			
**Number of visits during episode**			
Mean (min, max)	14 (1, 171)	12 (1, 171)	20 (1, 170)
Median (Q1, Q3)	12 (7, 18)	11 (7, 16)	18 (11, 26)
**Time between first and last evaluation, days**			
Mean (min, max)	44 (0, 692)	41 (0, 692)	59 (0, 488)
Median (Q1, Q3)	36 (23, 56)	35 (22, 52)	50 (31, 77)
**Maximum time between two consecutive visits, days**			
Mean (min, max)	8 (0, 90)	8 (0, 90)	8 (0, 89)
Median (Q1, Q3)	6 (5, 8)	6 (5, 9)	6 (5, 8)

Patients with operative care episodes were older, less likely to be female, and more likely to have commercial insurance and Medicare as compared to non-operative patients (all p<0.001). As expected, operative care episodes were more likely to include patients with primary body region of knee, foot/ankle, and shoulder and less likely to include patients with low back pain. Operative patients had a greater prevalence of high blood pressure, arthritis, and cancer at baseline compared to non-operative patients. However, there was no appreciable difference in the number of reported medical comorbidities and symptoms reported between operative and non-operative patients.

### National prevalence of no-shows

Overall, 73% of the population did not show for at least one appointment (Table 2). Patients who did not show for an appointment were more likely to be younger and female. Patients who presented with primary body region of general (87%), low back (76%), or neck pain (76%) were more likely to not show for an appointment as compared to patients who presented for other body regions. The same proportion of non-operative (73%) and operative patients did not show for an appointment (73%). A greater proportion of no-shows were for patients who had health care paid by Medicaid (85%) and workers’ compensation (79%) as compared to Medicare and commercial insurance. There were geographic variations in the proportion of patients with at least one no-show (range: 41–78%) (S2 Fig).

**Table 2:** Prevalence of no-shows within 1, 7, 12, and all visits.

	All patients N = 444,995	All appointments N = 323,502 (73%)	First 12 appointments N = 287,653 (65%)	First 7 appointments N = 229,156 (51%)	First appointment N = 21,915 (5%)
**Age**					
14–17	20203 (4.5%)	72.5%	65.1%	50.1%	3.7%
18–44	124625 (28.0%)	78.1%	70.8%	58.4%	5.5%
45–64	174211 (39.1%)	75.0%	66.5%	53.3%	5.2%
65+	125956 (28.3%)	64.2%	55.9%	42.4%	4.2%
**Sex**					
Male	182089 (40.9%)	70.2%	61.4%	48.2%	4.4%
Female	262906 (59.1%)	74.4%	66.9%	53.8%	5.3%
**Primary body region**					
Elbow/Wrist/Hand	12900 (2.9%)	68.1%	60.4%	47.5%	4.2%
Foot/Ankle	42263 (9.5%)	71.6%	62.7%	47.9%	3.6%
General	765 (0.2%)	87.1%	82.9%	71.0%	8.4%
Hip	32275 (7.3%)	69.3%	61.1%	47.0%	4.4%
Knee	85874 (19.3%)	70.1%	60.0%	46.5%	4.4%
Lumbar/SI	116298 (26.1%)	76.0%	70.0%	57.6%	6.0%
Neck	55397 (12.4%)	76.0%	69.5%	56.5%	5.3%
Other	29079 (6.5%)	68.6%	63.3%	53.0%	6.1%
Shoulder	70144 (15.8%)	72.4%	61.6%	47.7%	4.1%
**Post-operative**					
No	374791 (84.2%)	72.7%	66.7%	53.9%	5.1%
Yes	70204 (15.8%)	72.4%	53.9%	38.6%	3.8%
**Insurance provider**					
Commercial	257313 (57.8%)	72.1%	64.4%	51.1%	4.1%
Medicaid	44894 (10.1%)	84.7%	80.1%	70.3%	10.3%
Medicare	89760 (20.2%)	65.7%	57.2%	43.5%	4.5%
Other	8437 (1.9%)	70.9%	65.4%	56.0%	4.8%
Workers’ comp	44591 (10.0%)	78.6%	65.0%	50.0%	5.1%

Percentages enclosed in parentheses (%) were calculated using the entire analysis population, N = 444,995 patients. All other percentages shown were calculated using the corresponding row total.

The prevalence of patient no-shows was higher in patients 18–44 years of age (78%, 71%, 58%, 6%, respectively) as compared to patients 14–17 years of age (73%, 65%, 50%, and 4%, respectively), 45–65 years of age (75%, 67%, 53%, 5%, respectively) or patients greater than 65 years of age (64%, 55%, 42%, 4%, respectively (Table 2 and S3 Fig). Female patients were more likely not to show as compared to male patients. No-show prevalence was higher among non-operative care episodes as compared to operative care episodes regardless of number of visits. Across insurance providers, no-show rates were highest for patients with Medicaid and workers’ compensation.

### Prediction model development, performance, and results

**Fig 2:**
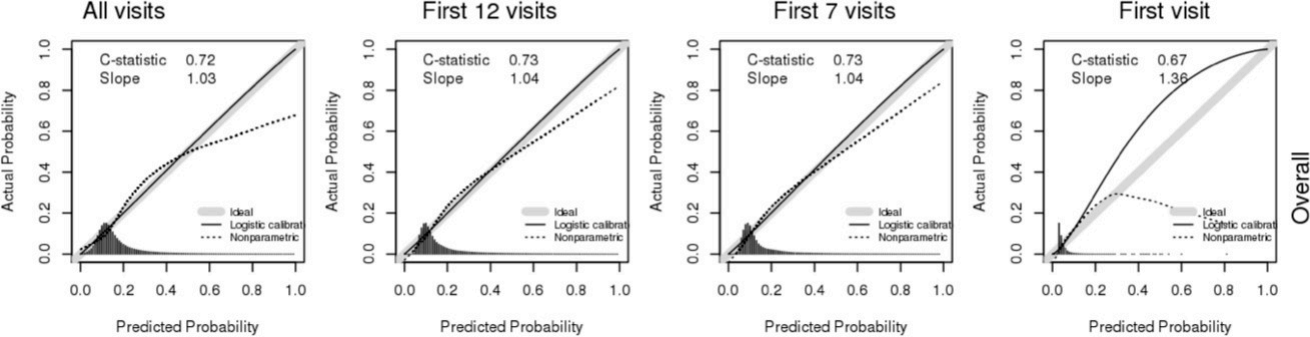
Model discrimination and calibration by cumulative visits.

A total of 333,747 patients were included in the training data, and 111,248 were included in the test dataset. The demographic and clinical characteristics of these patients are shown in S1 Table. There were no significant differences between the patients included in the training and testing datasets for demographic, clinical, encounter-related, and organizational variables. The prediction model had acceptable discrimination (c-statistic = 0.72) and very good calibration (calibration slope = 1.03) when all visits were included in the model (Fig 2).

**Fig 3:**
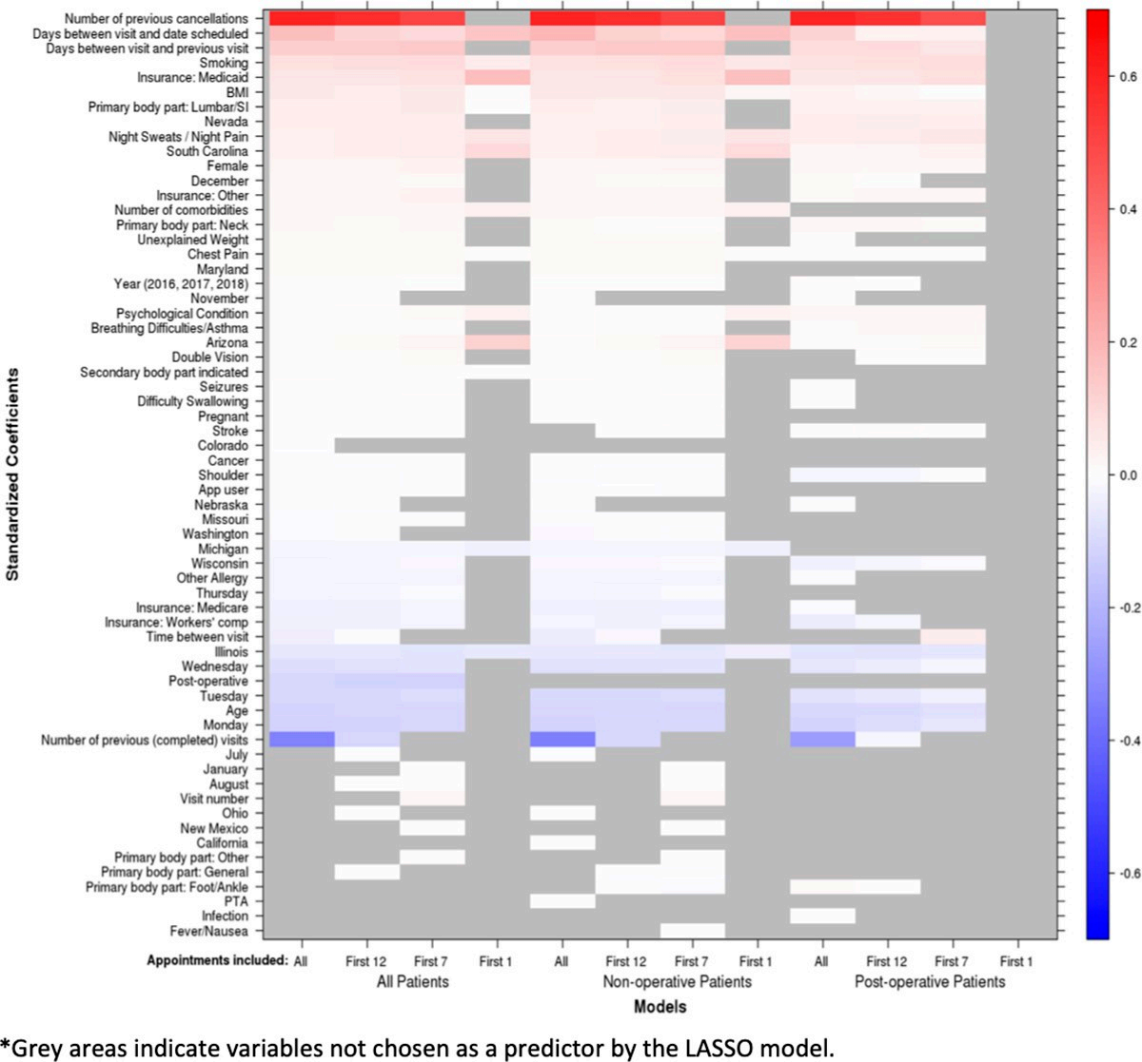
Variables predictive of a patient not showing up (red) and showing up (blue) for an appointment.

The strongest predictor of a patient *no-show* for all models (except for the first visit) was the number of previous cancelled visits (Fig 3). The other most common individual level characteristics that predicted a patient no-show included greater time between visit and date the visit was scheduled, greater time between current and prior visit, smoking status, Medicaid insurance, and higher BMI. Individual variables that predicted no-shows to a greater extent in the first visit included Medicaid insurance, night sweats/night pain, and number of comorbidities. Organizational factors that impacted prediction included time between date appointment was scheduled and date of appointment, time between current and prior appointment, clinic location (i.e., Nevada, South Carolina), month of year, and day of week.

Individual-level predictors of a patient *showing* for a visit included higher age, operative status, and insurance through workers’ compensation. Organizational-level predictors of showing for a visit included greater number of previously completed visits, day of week (Monday, Tuesday, Wednesday) and clinic location (i.e., Illinois). Predictors of showing for a visit were similar across visit number (except the first visit) and operative status. For the first visit, only clinic location (i.e., Illinois and Michigan) were predictive of patients showing for a visit.

### Sensitivity analysis

The distribution of the numbers of the visits was wide (i.e., 1–171). When we limited the number of visits to less than 12 and 7 visits, we found that the model discrimination, as measured by the c-statistic (AUC = 0.73 for both), and calibration, as measured by the calibration slope (slope = 1.04 for both), did not change (Fig 2). The prediction characteristics were robust until we used only the patient’s first visit, when the model discrimination decreased to an AUC of 0.67 and the model calibration increased to a slope of 1.36. The prediction characteristics were marginally better in non-operative patients when fewer visits were used and marginally better in operative patients when a greater number of visits were used in the model (S4 Fig).

Adolescents and adults have notably distinct causes of chronic pain. When results were stratified by age (adolescents vs. adults), we saw notable differences in the type of body region with which patients presented (S2 Table). Adolescents were more likely to present with foot/ankle and knee pain as compared to adults. Despite these differences in clinical presentation, predictive model characteristics, including the c-statistic and calibration slope, were similar across age groups (S5 Fig). Individual and demographic characteristics predictive of a patient no-show were similar across age categories (S6 Fig). Exclusion of insurance status did not appreciably change model calibration and discrimination or prediction characteristics (S7 and S8 Figs). Those individuals excluded from the analysis were younger, had lower BMI, were less likely to have ever smoked, and were more likely to have commercial insurance. In these individuals, the primary body region at presentation was more likely to be elbow/wrist/hand, foot/ankle, and general. They had fewer comorbidities and symptoms reports (S3 Table).

## Discussion

This is the first study, to our knowledge, to define the national prevalence of no-shows in the United States for seeking physical therapy care and to identify factors that predict patients likely to be no-shows. The notably high prevalence rate of no-shows creates inefficiencies for patients and the health care system through delayed non-pharmacological care, suboptimal dosing, and wasted resources. The risk prediction model we developed and validated has the potential to lead to the development of interventions that increase the efficiency of care and capacity to deliver non-pharmacologic treatments for MSK conditions. For example, patients identified as likely to no-show could be offered tele-health to replace or augment care delivered in a traditional clinic setting [35–38].

Contrary to our expectations, there were no obvious differences in predictors for non-operative and operative care episodes. We also investigated the models by different age categories and did not find obvious differences in predictors. Collectively these findings suggest wide application of predictive models for no-shows may be possible for those scheduled for physical therapy.

A number of studies have reported no-show rates ranging from 4% to 80% in multiple clinical areas, including family medicine [39–41], general medicine [42], pediatrics [43], dermatology [44, 45], cardiology [21, 46], neurology [21, 47], radiology [48, 49], gastroenterology [50, 51], ophthalmology [21, 52], endocrinology [21, 53], orthopedics [21], otolaryngology [21, 54, 55], plastic surgery [21, 56], pulmonary [21, 57], psychiatry [58], allergy [21, 57], oncology [59], rheumatology [21], urogynecology [60], and urology [18, 21, 61]. The median prevalence of no-shows is lower in adult and pediatric primary care settings (< 20%) and higher in specialty care such as cardiology (30%), endocrinology (36%) and physiotherapy/physical therapy (57%). There are a limited number of studies reporting no-show rates or no-show prediction algorithms for physical therapy. Two studies were conducted in the same population in Nigeria, using data from an outpatient physiotherapy clinic [62, 63]. The authors reported no-show rates between 57% and 79%. They included gender, comorbidities, distance to facility, and month of appointment in their prediction model. One additional study–conducted in the United States–reported a prediction model that was developed using decision trees [64].

Across all appointments, we found that 73% of physical therapy patients did not show for at least one appointment. Across clinics, states, and providers no-show rates ranged from 15–31% reflecting the daily impact of no-shows on efficiency of care delivery. We suspect that the variation in no-show rates across specialties can be attributed to the timing of care. That is, patients receive physical therapy in a condensed period of time (i.e., multiple visits over a few weeks) while care in other specialties can be delivered over a long period of time (i.e., few visits over multiple months). In our study, adolescents had a slightly lower rate of no-shows as compared to young and middle-aged adults. Prior research in different patient populations has suggested that pediatric no-shows may be due to parents forgetting the appointment [65], parents thinking their child’s health has improved [65], chaotic homes [66], or socioeconomic [56] factors. These factors may not be as strong of an influence for adolescents seeking care for MSK conditions. At the state level, we saw variation in the proportion of patients who missed an appointment. This variation may be attributable to state level difference in insurance coverage, differing perceptions about physical therapy, and/or the number of providers in the state. Our findings add to the existing literature by being the first study to report the national prevalence of no-shows among patients seeking physical therapy for MSK conditions.

Non-pharmacological pain management, such as delivered during physical therapy care, has been recommended as first-line options in recent clinical practice guidelines, but current practice models do not adequately meet that charge. For example, opioids are frequently prescribed for back pain in the United States [3]. In contrast, there were large decreases in short and long term opioid use when patients had non-pharmacological care first for low back pain from physical therapists, chiropractors, and acupuncturists [10]. Patient no-shows represent a significant barrier to improving rates of non-pharmacological care as a first-line option. This is an important barrier to address because multiple observational studies indicate that exposure to physical therapy can prevent long-term opioid use and decrease receipt of low-value care (i.e., advanced diagnostic imaging and spinal injections) for common MSK conditions [7–9, 67].

Interventions that have been implemented previously to reduce no-shows include reminder letters and a centralized phone system to schedule appointments for all clinics using one toll-free number [18]. Another option that is currently relevant due to COVID-19 restrictions would be to offer telehealth delivery [35]. These strategies would involve systematically identifying individuals that are at highest risk for a patient no-show. A final option that would not involve intervention at the individual patient level would be to overschedule appointments for clinics that have organization factors that suggest the likelihood of higher no-show rates. This strategy would have to be carefully implemented to avoid over-crowding or long waits at clinical sites.

There are notable strengths to our study. This cohort is a general US population receiving care from a single physical therapy provider with clinics that include urban and rural locations, including representation from geographic regions with high opioid use. Additionally, the results are highly generalizable due to the broad age range and geography, and wide heterogeneity of MSK conditions considered. Another strength is that acceptable predictive accuracy was achieved using a pragmatic set of variables, which may enable implementation across EHRs in other systems. There are also limitations to note with our approach. We did not utilize formal methods (e.g., Delphi method, focus groups) when we consulted with clinical providers and the company’s administrators to identify predictors of no-shows. After this conversation though, we did include all available variables present in the EHR. The other broad category of limitations involved missing data, or the absence of variables theorized to impact patient no-shows. As these data involve real-world patients, they exemplify the challenges of using administrative data for clinical research. Specifically, we had considerable missing data for patient age, height, weight, and BMI. It can be postulated that patients may be reticent to supply this information for behavioral or social reasons [68, 69]. Although these data were drawn from a national physical therapy provider, the provider does not have clinics in all states, potentially impacting the generalizability of our results. However, as previously mentioned, there is considerable overlap between the state in which a clinic is located and states with a high burden of opioid overuse [70]. In addition, these data lack granular information regarding health insurance policies, limiting our ability to control for deductibles and copayment. These missing data may be important because socioeconomic status likely influences access to care and subsequent treatment responses for MSK conditions [71]. As an example, Medicaid was a predictor of no-shows in our study. Baseline patient-reported pain severity and measures of psychological distress were also unavailable and may be important to consider for improving no-show prediction or building engagement strategies that would increase attendance. Broader health utilization information identifying other providers being seen concurrently with the PT episode was unknown and could not be included in the analysis. Finally, potentially relevant predictor variables including race, lead time, date of cancellation, and distance to clinic were missing, not collected, or unavailable for analysis.

This study also provided direction for future research. Broader health utilization information identifying other providers being seen concurrently with the PT episode was unknown and could not be included in the analysis. For example, potentially relevant predictor variables including race, lead time, date of cancellation, acute or chronic pain and distance to clinic were missing, not collected, or unavailable for analysis. In future studies these variables could be incorporated into the existing predictor model to see if accuracy is improved. Also, the current study did not directly incorporate the patient perspective and future research could use mixed-methods approaches to further explore reasons for no-show appointments. Finally, future research that tests this predictive model in other physical therapy settings would help to further inform its validity and determine if setting specific modifications need to be made to the model.

In conclusion, we found a high rate of no-shows for patients with MSK conditions making an appointment with a physical therapy provider. The no-show rate varied by age, gender, insurance status, and other individual factors (e.g., smoking status, BMI, and number of comorbidities). We developed a novel prediction algorithm that performed similarly across non-operative and operative status with discrimination and calibration stabilized in as few as four visits. Future research should focus on testing intervention strategies that are tailored to patients at high for no-shows and either decrease no-shows or expand access through coordinated use of tele-health.

## Supporting information

S1 Table Baseline characteristics comparing training and test datasets. (PDF)

S2 Table Baseline characteristics by age group (i.e., adolescents and adults). (PDF)

S3 Table Baseline characteristics of the study population excluded and included in the study. (PDF)

S1 Fig Study population included and excluded in the analyses. (TIF)

S2 Fig Proportion of patients in each state who canceled at least 1 appointment. (TIF)

S3 Fig Cancellation rate by visit number and age group. (TIF)

S4 Fig Model discrimination and calibration by cumulative visits and operative status. (TIF)

S5 Fig Model discrimination and calibration stratified by age category. (TIF)

S6 Fig Characteristics predictive of patient no-show by age category. (TIF)

S7 Fig Model discrimination and calibration stratified by age category. (TIF)

S8 Fig Characteristics predictive of patient no-show by age category, excluding insurance status. (TIF)
