# Supplementary material for: Prevalence and predictors of no-shows to physical therapy for musculoskeletal conditions
Source: PLoS One. 2021 May 28;16(5):e0251336. doi: 10.1371/journal.pone.0251336 (PMC8162651; doi:10.1371/journal.pone.0251336)
Supplement: S1 Table — (PDF) [file pone.0251336.s001.pdf]

	Dataset assignment			
	Overall<br>N=444,995	Training<br>N=333,747	Test<br>N=111,248	P-value
**Patient-level characteristics**				
**Age (years)**				0.939
Mean (min, max)	53 (14, 117)	53 (14, 116)	53 (14, 117)	
Median (Q1, Q3)	54 (39, 67)	54 (39, 67)	54 (39, 67)	
**Female**	262906 (59.1%)	197233 (59.1%)	65673 (59.0%)	0.709
**Pregnant**	1749/262906 (0.7%)	1306/197233 (0.7%)	443/65673 (0.7%)	0.735
**Height (inches)**				0.306
Mean (min, max)	67 (36, 84)	67 (38, 84)	67 (36, 84)	
Median (Q1, Q3)	66 (64, 70)	66 (64, 70)	66 (64, 70)	
**Weight (pounds)**				0.026
Mean (min, max)	182 (54, 400)	182 (57, 400)	182 (54, 400)	
Median (Q1, Q3)	175 (149, 208)	175 (149, 208)	175 (150, 208)	
**BMI (kg/m^2)**				0.02
Mean (min, max)	29 (11, 75)	29 (11, 75)	29 (11, 71)	
Median (Q1, Q3)	27 (24, 32)	27 (24, 32)	27 (24, 32)	
**Ever Smoker**	65275 (14.7%)	48950 (14.7%)	16325 (14.7%)	0.95
**Insurance provider**				0.25
Commercial	257313 (57.8%)	193259 (57.9%)	64054 (57.6%)	
Medicaid	44894 (10.1%)	33555 (10.1%)	11339 (10.2%)	
Medicare	89760 (20.2%)	67299 (20.2%)	22461 (20.2%)	
Other	8437 (1.9%)	6326 (1.9%)	2111 (1.9%)	
Workers' Comp	44591 (10.0%)	33308 (10.0%)	11283 (10.1%)	
**Therapist type**				0.02
PT	436007 (98.0%)	327103 (98.0%)	108904 (97.9%)	
PTA	8988 (2.0%)	6644 (2.0%)	2344 (2.1%)	
**Number of providers at the clinic**				0.74
Mean (min, max)	2 (0, 15)	2 (0, 15)	2 (0, 15)	

	Dataset assignment			
	Overall<br>N=444,995	Training<br>N=333,747	Test<br>N=111,248	P-value
Median (Q1, Q3)	2 (1, 3)	2 (1, 3)	2 (1, 3)	
**Clinical characteristics**				
Primary body region				0.61
Elbow/Wrist/Hand	12900 (2.9%)	9685 (2.9%)	3215 (2.9%)	
Foot/Ankle	42263 (9.5%)	31663 (9.5%)	10600 (9.5%)	
General	765 (0.2%)	583 (0.2%)	182 (0.2%)	
Hip	32275 (7.3%)	24364 (7.3%)	7911 (7.1%)	
Knee	85874 (19.3%)	64394 (19.3%)	21480 (19.3%)	
Lumbar/SI	116298 (26.1%)	87148 (26.1%)	29150 (26.2%)	
Neck	55397 (12.4%)	41525 (12.4%)	13872 (12.5%)	
Shoulder	70144 (15.8%)	52521 (15.7%)	17623 (15.8%)	
Other	29079 (6.5%)	21864 (6.6%)	7215 (6.5%)	
Chronic injury	81354 (18.3%)	61023 (18.3%)	20331 (18.3%)	0.95
**Comorbidities**				
Arthritis	165354 (37.2%)	124030 (37.2%)	41324 (37.1%)	0.92
High blood pressure	145655 (32.7%)	109078 (32.7%)	36577 (32.9%)	0.23
Breathing difficulties/ Asthma	65205 (14.7%)	48930 (14.7%)	16275 (14.6%)	0.80
Diabetes	57127 (12.8%)	42757 (12.8%)	14370 (12.9%)	0.36
Heart condition	47506 (10.7%)	35786 (10.7%)	11720 (10.5%)	0.08
Osteoporosis	39395 (8.9%)	29689 (8.9%)	9706 (8.7%)	0.08
Cancer	36491 (8.2%)	27302 (8.2%)	9189 (8.3%)	0.40
Psychological condition	28134 (6.3%)	21097 (6.3%)	7037 (6.3%)	0.96
Chest pain	22093 (5.0%)	16635 (5.0%)	5458 (4.9%)	0.30
Kidney condition	18458 (4.1%)	13860 (4.2%)	4598 (4.1%)	0.78
Stroke	14529 (3.3%)	10965 (3.3%)	3564 (3.2%)	0.18

	Dataset assignment			
	**Overall N=444,995**	**Training N=333,747**	**Test N=111,248**	**P-value**
**Symptom reports**				
Night sweats/night pain	71316 (16.0%)	53537 (16.0%)	17779 (16.0%)	0.64
Ringing in your ears	60499 (13.6%)	45498 (13.6%)	15001 (13.5%)	0.21
Fracture	43073 (9.7%)	32220 (9.7%)	10853 (9.8%)	0.32
Difficulty swallowing	14906 (3.3%)	11146 (3.3%)	3760 (3.4%)	0.52
**Number of comorbidities**				0.71
Mean (min, max)	1 (0, 11)	1 (0, 11)	1 (0, 11)	
Median (Q1, Q3)	1 (0, 2)	1 (0, 2)	1 (0, 2)	
**Number of symptoms reported**				0.78
Mean (min, max)	0 (0, 4)	0 (0, 4)	0 (0, 4)	
Median (Q1, Q3)	0 (0, 1)	0 (0, 1)	0 (0, 1)	
**Visits**				
Number of visits during episode				0.79
Mean (min, max)	14 (1, 171)	14 (1, 171)	14 (1, 153)	
Median (Q1, Q3)	12 (7, 18)	11 (7, 18)	12 (7, 18)	
Time between first and last evaluation, days				0.76
Mean (min, max)	44 (0, 692)	44 (0, 692)	44 (0, 491)	
Median (Q1, Q3)	36 (23, 56)	36 (23, 56)	36 (23, 56)	
Maximum time between two consecutive visits, days				0.14
Mean (min, max)	8 (0, 90)	8 (0, 90)	8 (0, 87)	
Median (Q1, Q3)	6 (5, 8)	6 (5, 8)	6 (5, 8)	
