# Supplementary material for: Prevalence and predictors of no-shows to physical therapy for musculoskeletal conditions
Source: PLoS One. 2021 May 28;16(5):e0251336. doi: 10.1371/journal.pone.0251336 (PMC8162651; doi:10.1371/journal.pone.0251336)
Supplement: S2 Table — (PDF) [file pone.0251336.s002.pdf]

	Overall N=444,995	Age 14-17 N=20,203	Age 18+ N=424,792	P-value
**Patient-level characteristics**				
**Age (years)**				<.001
Mean (min, max)	53 (14, 117)	16 (14, 18)	54 (18, 117)	
Median (Q1, Q3)	54 (39, 67)	16 (15, 17)	56 (42, 67)	
**Female**	262906 (59.1%)	10722 (53.1%)	252184 (59.4%)	<.001
**Pregnant**	1749/262906 (0.7%)	7/10722 (0.1%)	1742/252184 (0.7%)	<.001
**Height (inches)**				<.001
Mean (min, max)	67 (36, 84)	67 (40, 83)	67 (36, 84)	
Median (Q1, Q3)	66 (64, 70)	67 (64, 70)	66 (64, 70)	
**Weight (pounds)**				<.001
Mean (min, max)	182 (54, 400)	149 (61, 400)	183 (54, 400)	
Median (Q1, Q3)	175 (149, 208)	140 (125, 165)	178 (150, 210)	
**BMI (kg/m^2)**				<.001
Mean (min, max)	29 (11, 75)	23 (11, 61)	29 (11, 75)	
Median (Q1, Q3)	27 (24, 32)	22 (20, 25)	28 (24, 32)	
**Ever smoker**	65275 (14.7%)	193 (1.0%)	65082 (15.3%)	<.001
**Insurance provider**				<.001
Commercial	257313 (57.8%)	15280 (75.6%)	242033 (57.0%)	
Medicaid	44894 (10.1%)	3520 (17.4%)	41374 (9.7%)	
Medicare	89760 (20.2%)	1 (0.0%)	89759 (21.1%)	
Other	8437 (1.9%)	876 (4.3%)	7561 (1.8%)	
Workers' Comp	44591 (10.0%)	526 (2.6%)	44065 (10.4%)	
**Therapist type**				0.36
PT	436007 (98.0%)	19813 (98.1%)	416194 (98.0%)	
PTA	8988 (2.0%)	390 (1.9%)	8598 (2.0%)	
**Number of providers at the clinic**				<.001
Mean (min, max)	2 (0, 15)	3 (0, 15)	2 (0, 15)	
Median (Q1, Q3)	2 (1, 3)	2 (1, 3)	2 (1, 3)	
**Clinical characteristics**				
Primary body region				<.001
Elbow/wrist/hand	12900 (2.9%)	710 (3.5%)	12190 (2.9%)	

Foot/Ankle	42263 (9.5%)	3596 (17.8%)	38667 (9.1%)	
General	765 (0.2%)	12 (0.1%)	753 (0.2%)	
Hip	32275 (7.3%)	1850 (9.2%)	30425 (7.2%)	
Knee	85874 (19.3%)	6394 (31.6%)	79480 (18.7%)	
Lumbar/SI	116298 (26.1%)	3499 (17.3%)	112799 (26.6%)	
Neck	55397 (12.4%)	1162 (5.8%)	54235 (12.8%)	
Shoulder	70144 (15.8%)	2464 (12.2%)	67680 (15.9%)	
Other	29079 (6.5%)	516 (2.6%)	28563 (6.7%)	
Chronic injury	81354 (18.3%)	2911 (14.4%)	78443 (18.5%)	<.001
**Comorbidities**				
Arthritis	165354 (37.2%)	186 (0.9%)	165168 (38.9%)	<.001
High blood pressure	145655 (32.7%)	126 (0.6%)	145529 (34.3%)	<.001
Breathing difficulties/asthma	65205 (14.7%)	2821 (14.0%)	62384 (14.7%)	0.005
Diabetes	57127 (12.8%)	131 (0.6%)	56996 (13.4%)	<.001
Heart condition	47506 (10.7%)	234 (1.2%)	47272 (11.1%)	<.001
Osteoporosis	39395 (8.9%)	21 (0.1%)	39374 (9.3%)	<.001
Cancer	36491 (8.2%)	53 (0.3%)	36438 (8.6%)	<.001
Psychological condition	28134 (6.3%)	486 (2.4%)	27648 (6.5%)	<.001
Chest pain	22093 (5.0%)	338 (1.7%)	21755 (5.1%)	<.001
Kidney condition	18458 (4.1%)	106 (0.5%)	18352 (4.3%)	<.001
Stroke	14529 (3.3%)	20 (0.1%)	14509 (3.4%)	<.001
**Symptom Reports**				
Night sweats/night pain	71316 (16.0%)	444 (2.2%)	70872 (16.7%)	<.001
Ringing in your ears	60499 (13.6%)	475 (2.4%)	60024 (14.1%)	<.001
Fracture	43073 (9.7%)	2283 (11.3%)	40790 (9.6%)	<.001
Difficulty swallowing	14906 (3.3%)	103 (0.5%)	14803 (3.5%)	<.001
**Number of comorbidities**				<.001
Mean (min, max)	1 (0, 11)	0 (0, 6)	1 (0, 11)	
Median (Q1, Q3)	1 (0, 2)	0 (0, 0)	1 (0, 2)	
**Number of symptoms reported**				<.001
Mean (min, max)	0 (0, 4)	0 (0, 4)	0 (0, 4)	

Median (Q1, Q3)	0 (0, 1)	0 (0, 0)	0 (0, 1)	
**Visits**				
Number of visits during episode				<.001
Mean (min, max)	14 (1, 171)	13 (1, 120)	14 (1, 171)	
Median (Q1, Q3)	12 (7, 18)	11 (7, 16)	12 (7, 18)	
Time between first and last evaluation, days				0.65
Mean (min, max)	44 (0, 692)	44 (0, 532)	44 (0, 692)	
Median (Q1, Q3)	36 (23, 56)	35 (22, 54)	36 (23, 56)	
Maximum time between two consecutive visits, days				<.001
Mean (min, max)	8 (0, 90)	8 (0, 86)	8 (0, 90)	
Median (Q1, Q3)	6 (5, 8)	7 (5, 9)	6 (5, 8)	
