# Supplementary material for: Prevalence and predictors of no-shows to physical therapy for musculoskeletal conditions
Source: PLoS One. 2021 May 28;16(5):e0251336. doi: 10.1371/journal.pone.0251336 (PMC8162651; doi:10.1371/journal.pone.0251336)
Supplement: S3 Table — (PDF) [file pone.0251336.s003.pdf]

	Excluded N=112,346	Included N=444,995
**Patient Level Characteristics**		
**Age (years)**		
Mean (min, max)	45 (0, 104)	53 (14, 117)
Median (Q1, Q3)	48 (26, 62)	54 (39, 67)
**Female**	67205 (59.8%)	262906 (59.1%)
**Pregnant**	899/67205 (1.3%)	1749/262906 (0.7%)
**Height (inche)**		
Mean (min, max)	61 (13, 720)	67 (36, 84)
Median (Q1, Q3)	62 (58, 65)	66 (64, 70)
**Weight (pounds)**		
Mean (min, max)	121 (1, 972)	182 (54, 400)
Median (Q1, Q3)	106 (85, 130)	175 (149, 208)
**BMI (kg/m^2)**		
Mean (min, max)	22 (0, 308)	29 (11, 75)
Median (Q1, Q3)	20 (18, 23)	27 (24, 32)
**Ever Smoker**	11766 (10.5%)	65275 (14.7%)
**Insurance provider**		
Commercial	71552 (63.7%)	257313 (57.8%)
Medicaid	12293 (10.9%)	44894 (10.1%)
Medicare	15569 (13.9%)	89760 (20.2%)
Other	2977 (2.6%)	8437 (1.9%)
Workers' Comp	9955 (8.9%)	44591 (10.0%)
**Therapist type**		
PT	109447 (97.4%)	436007 (98.0%)
PTA	2899 (2.6%)	8988 (2.0%)
**Number of Providers at the Clinic**		
Mean (min, max)	3 (0, 15)	2 (0, 15)
Median (Q1, Q3)	2 (1, 3)	2 (1, 3)
**Clinical Characteristics**		
**Primary body region**		
Elbow/Wrist/Hand	5033 (4.5%)	12900 (2.9%)
Foot/Ankle	13896 (12.4%)	42263 (9.5%)
General	8454 (7.5%)	765 (0.2%)
Hip	8354 (7.4%)	32275 (7.3%)
Knee	23612 (21.0%)	85874 (19.3%)
Lumbar/SI	21437 (19.1%)	116298 (26.1%)
Neck	10820 (9.6%)	55397 (12.4%)
Other	5843 (5.2%)	29079 (6.5%)
Shoulder	14897 (13.3%)	70144 (15.8%)
Chronic injury	23421 (20.8%)	81354 (18.3%)

**Comorbidities**		
Arthritis	29828 (26.6%)	165354 (37.2%)
High blood pressure	26093 (23.2%)	145655 (32.7%)
Breathing difficulties/ Asthma	14266 (12.7%)	65205 (14.7%)
Diabetes	10290 (9.2%)	57127 (12.8%)
Heart condition	8775 (7.8%)	47506 (10.7%)
Osteoporosis	6882 (6.1%)	39395 (8.9%)
Cancer	6516 (5.8%)	36491 (8.2%)
Psychological condition	5822 (5.2%)	28134 (6.3%)
Chest pain	4101 (3.7%)	22093 (5.0%)
Kidney condition	3367 (3.0%)	18458 (4.1%)
Stroke	2568 (2.3%)	14529 (3.3%)
**Symptom Reports**		
Night sweats/ Night pain	13018 (11.6%)	71316 (16.0%)
Ringing in your ears	11132 (9.9%)	60499 (13.6%)
Fracture	10496 (9.3%)	43073 (9.7%)
Difficulty swallowing	2738 (2.4%)	14906 (3.3%)
**Number of comorbidities**		
Mean (min, max)	1 (0, 11)	1 (0, 11)
Median (Q1, Q3)	0 (0, 2)	1 (0, 2)
**Number of symptoms reported**		
Mean (min, max)	0 (0, 4)	0 (0, 4)
Median (Q1, Q3)	0 (0, 1)	0 (0, 1)
**Visits**		
**Number of visits during episode**		
Mean (min, max)	13 (1, 193)	14 (1, 171)
Median (Q1, Q3)	10 (6, 16)	12 (7, 18)
**Time between first and last evaluation, days**		
Mean (min, max)	43 (0, 644)	44 (0, 692)
Median (Q1, Q3)	35 (20, 56)	36 (23, 56)
**Maximum time between two consecutive visits, days**		
Mean (min, max)	8 (0, 90)	8 (0, 90)
Median (Q1, Q3)	6 (5, 9)	6 (5, 8)
