# Supplementary figures and images for: Prevalence and predictors of no-shows to physical therapy for musculoskeletal conditions
Source: PLoS One. 2021 May 28;16(5):e0251336. doi: 10.1371/journal.pone.0251336 (PMC8162651; doi:10.1371/journal.pone.0251336)

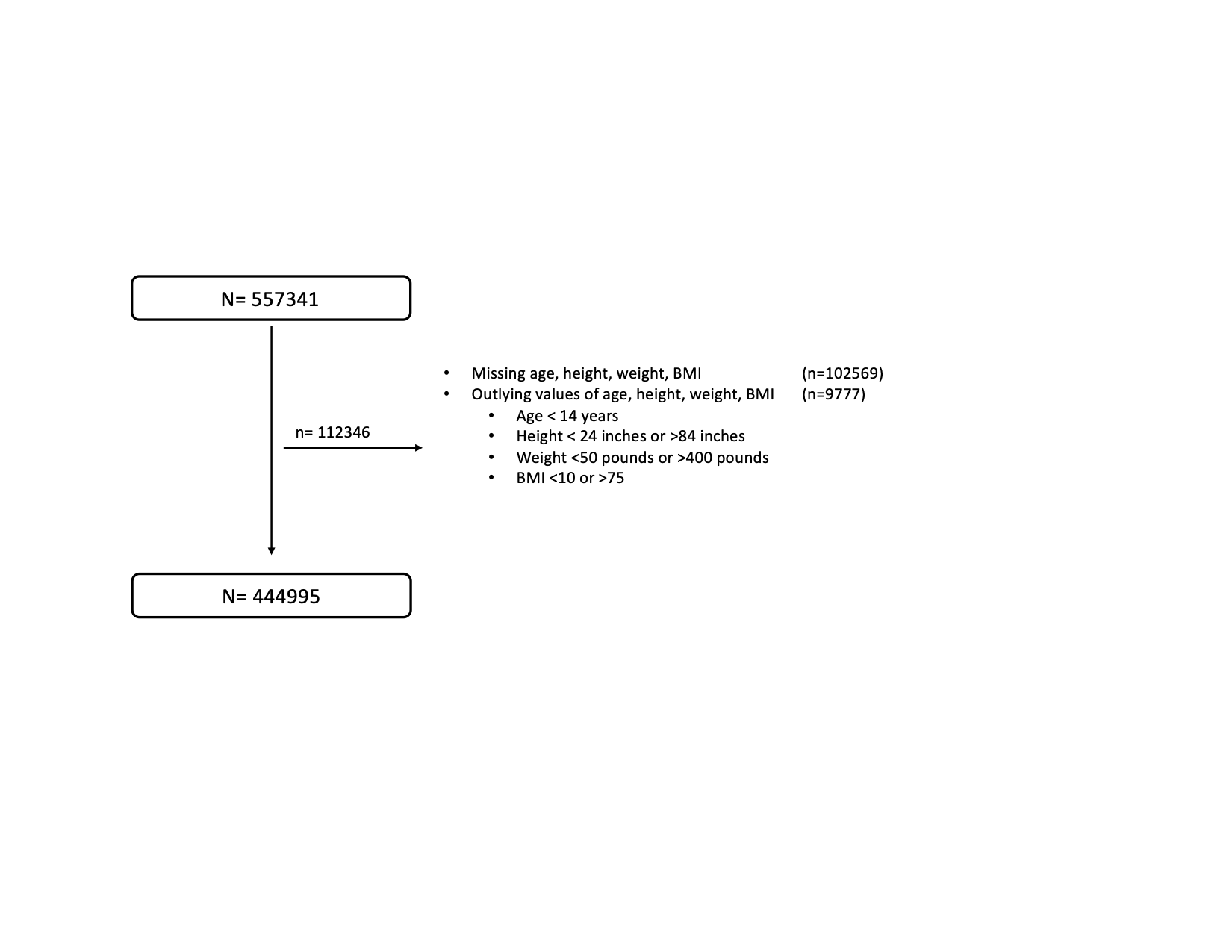

Supplement: S1 Fig — (TIF) [file pone.0251336.s004.tif]

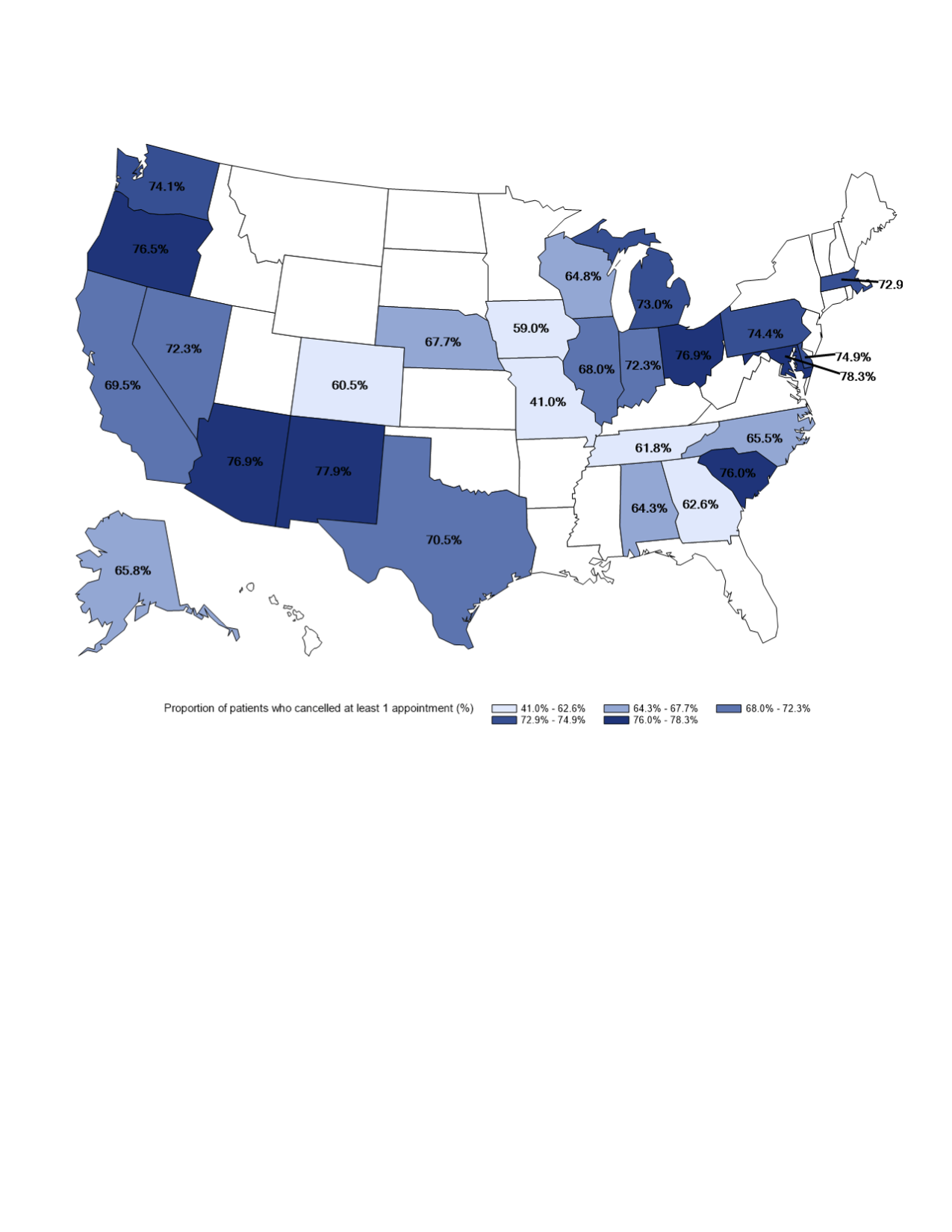

Supplement: S2 Fig — (TIF) [file pone.0251336.s005.tif]

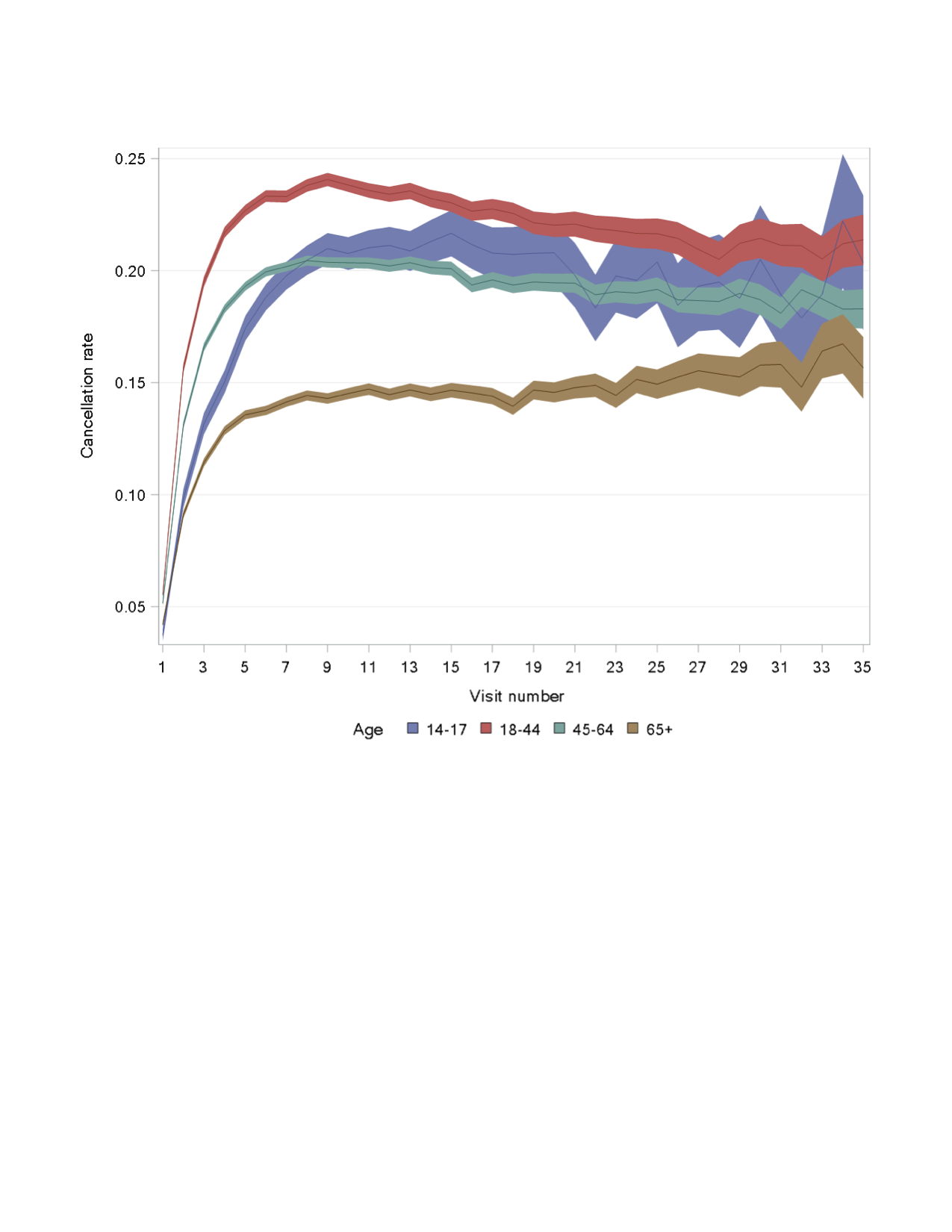

Supplement: S3 Fig — (TIF) [file pone.0251336.s006.tif]

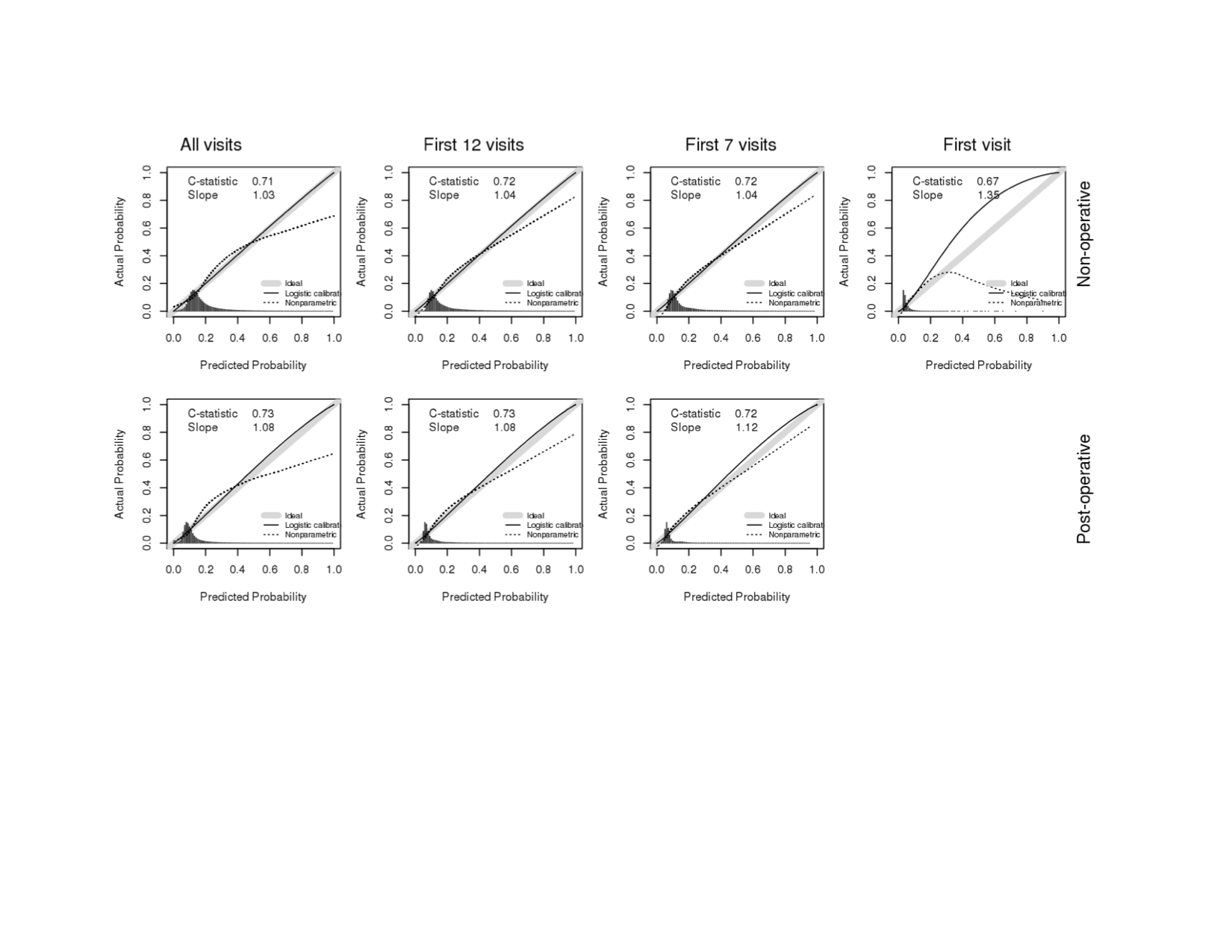

Supplement: S4 Fig — (TIF) [file pone.0251336.s007.tif]

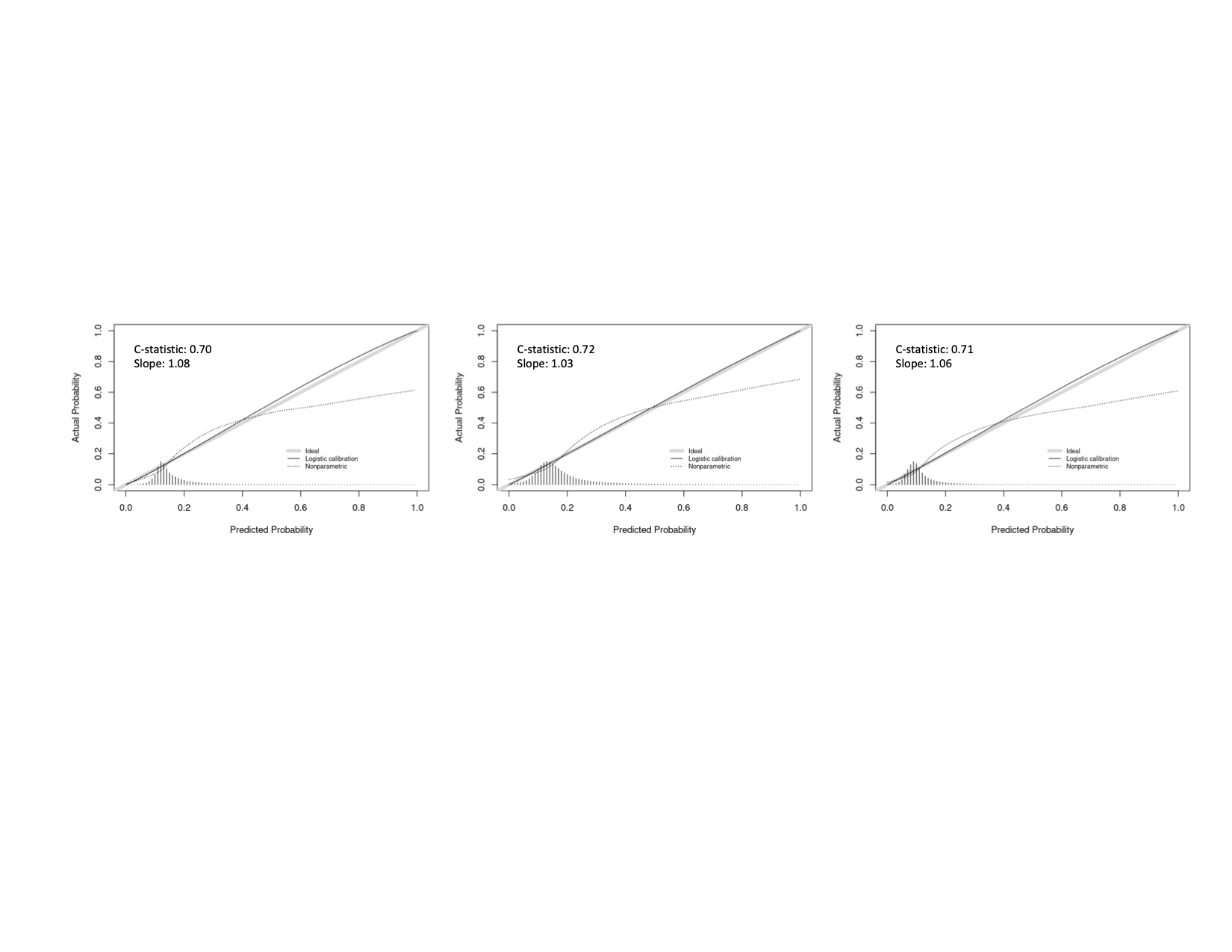

Supplement: S5 Fig — (TIF) [file pone.0251336.s008.tif]

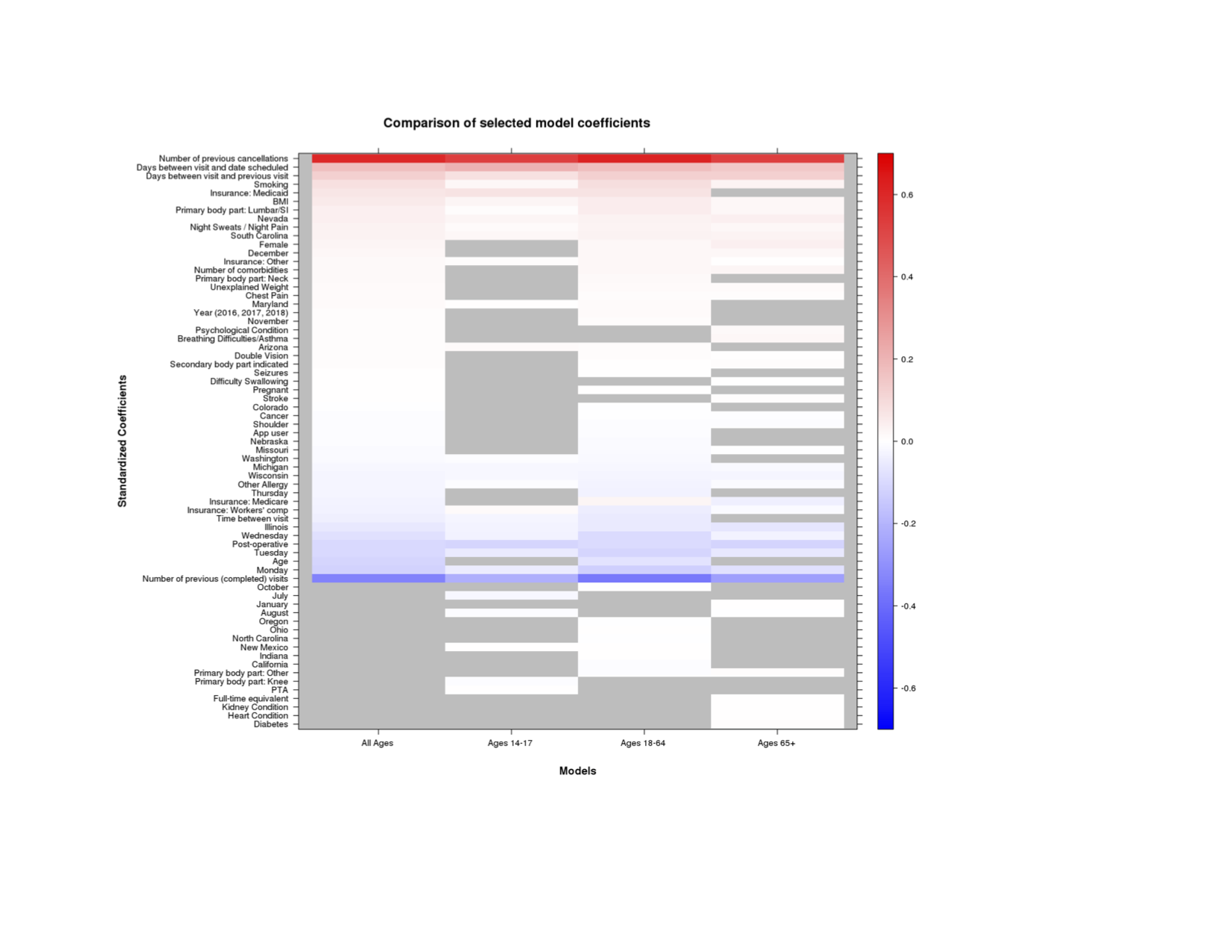

Supplement: S6 Fig — (TIF) [file pone.0251336.s009.tif]

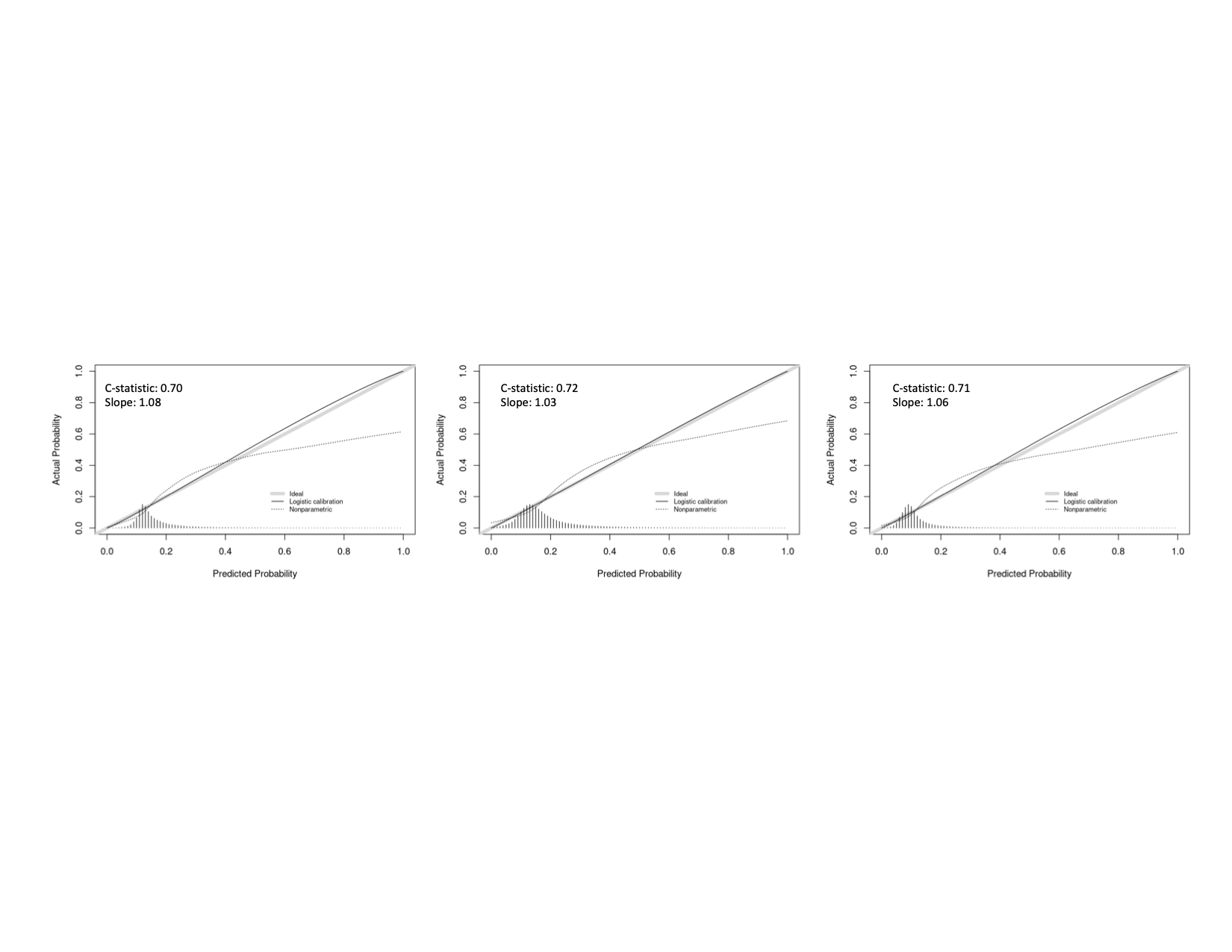

Supplement: S7 Fig — (TIF) [file pone.0251336.s010.tif]

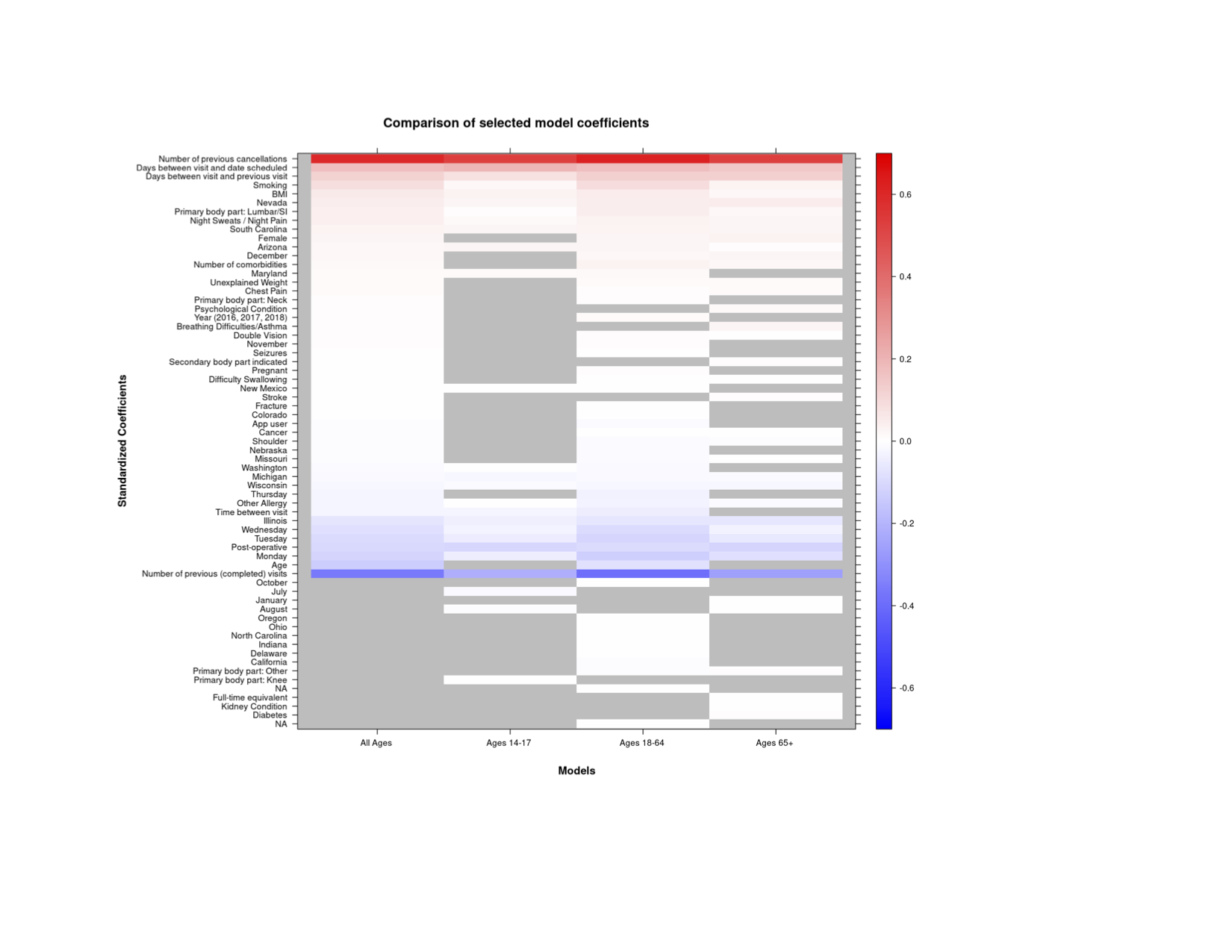

Supplement: S8 Fig — (TIF) [file pone.0251336.s011.tif]
